# Hepatitis E virus in the Kathmandu Valley: Insights from a representative longitudinal serosurvey

**DOI:** 10.1371/journal.pntd.0012375

**Published:** 2024-08-05

**Authors:** Nishan Katuwal, Melina Thapa, Sony Shrestha, Krista Vaidya, Isaac I. Bogoch, Rajeev Shrestha, Jason R. Andrews, Dipesh Tamrakar, Kristen Aiemjoy

**Affiliations:** 1 Research and Development Division, Dhulikhel Hospital Kathmandu University Hospital, Dhulikhel, Nepal; 2 Center for Infectious Disease Research and Surveillance, Dhulikhel Hospital, Kathmandu University Hospital, Dhulikhel, Nepal; 3 Division of Epidemiology, Department of Public Health Sciences, University of California Davis School of Medicine, Sacramento, California, United States of America; 4 Department of Medicine, University of Toronto, Toronto, Canada; 5 Department of Pharmacology, Kathmandu University School of Medical Sciences, Dhulikhel, Nepal; 6 Division of Infectious Diseases and Geographic Medicine, Stanford University School of Medicine, Stanford, California, United States of America; 7 Department of Community Medicine, Kathmandu University School of Medical Sciences, Dhulikhel, Nepal; 8 Department of Microbiology and Immunology, Mahidol University Faculty of Tropical Medicine, Bangkok, Thailand; Tufts Medical Center, UNITED STATES OF AMERICA

## Abstract

**Background:**

Hepatitis-E virus (HEV), an etiologic agent of acute inflammatory liver disease, is a significant cause of morbidity and mortality in South Asia. HEV is considered endemic in Nepal; but data on population-level infection transmission is sparse.

**Methods:**

We conducted a longitudinal serosurvey in central Nepal to assess HEV exposure. At each visit, capillary blood samples were collected and analyzed for the presence of anti-HEV IgG antibodies. The study took place between February 2019 and April 2021, with up to 4 visits per participant approximately 6 months apart.

**Results:**

We collected 2513 samples from 923 participants aged 0–25 years, finding a seroprevalence of 4.8% and a seroincidence rate of 10.9 per 1000 person-years. Young adults and individuals consuming surface water faced the highest incidence of infection. Geospatial analysis identified potential HEV clusters, suggesting a need for targeted interventions.

**Significance:**

Our findings demonstrate that HEV is endemic in Nepal and that the risk of infection increases with age.

## Author summary

Hepatitis-E virus (HEV) is an infectious cause of acute liver disease and is primarily transmitted through contaminated food and water. It has emerged as a leading cause of acute clinical hepatitis in South Asia. Pregnant women face the highest risk of complications and mortality. To better understand the impact of HEV in Nepal, we conducted a two-year cohort study in the Kathmandu Valley region. We collected dried blood spots from participants every 6 months and tested the samples to detect past and new infections of HEV. Our study, involving 923 individuals, revealed that 4.8% had IgG antibodies against HEV, indicating past exposure. Each year, about 11 in 1000 people between the ages of 1 and 25 years are newly exposed to the virus. The highest infection rates were among young adults and those using untreated surface water. Our study mapped the distribution of HEV, identifying specific areas with higher infection rates.

## Introduction

Hepatitis E Virus (HEV), first identified in 1983, has emerged as the leading cause of acute clinical hepatitis in South Asia [1]. The overall mortality rate associated with HEV is 1–4% [2]. Immunocompromised individuals and pregnant women face the highest risk for complications and death, with mortality rates reaching up to 20% among pregnant women in their third trimester [3]. The virus is primarily transmitted through food and water contaminated with infected fecal material, disproportionately affecting individuals in locations lacking improved sanitation systems [2,4].

HEV is estimated to cause approximately 20 million new infections annually, leading to around 3.3 million symptomatic cases and 70,000 deaths [5]. However, these figures may be underestimated due to limited surveillance capacity and suboptimal access to laboratory diagnostics, which often leave many infections, especially pauci-symptomatic and subclinical cases, undetected and under-reported [6].

Sero-epidemiology provides a valuable tool to augment clinical surveillance of HEV, particularly when diagnostics and reporting systems are limited [7]. *Sero-epidemiology* also offers insights into exposure that are not biased by access to health care and care-seeking behaviors [8]. Antibody responses to the four recognized HEV genotypes are similar [2]. IgG antibody responses peak 2–6 weeks post-infection [9,10]. A study of confirmed HEV patients in Nepal found that IgG responses decayed substantially in the first 6 months but remained elevated above a seropositivity threshold for at least 14 months [11].

Nepal has experienced repeated outbreaks of HEV with sporadic cases of acute hepatitis between outbreaks [12]. A study among individuals age 1–93 years seeking orthopedic care in Kathmandu between 2010 and 2012 found an IgG seroprevalence of 47.1% [13]. Among healthy blood donors age 18–55 years in Kathmandu in 2014, the age-adjusted seroprevalence of HEV was 3.2% for IgM and 8.3% for IgG [14]. A study conducted after the 2015 earthquake among blood donors residing in earthquake-affected areas found a HEV seroprevalence of 3.2% for IgM and 41.9% for IgG [15]. Most previous seroprevalence studies were conducted using convenience samples (healthy blood donors or individuals seeking care in a hospital setting), and it is unknown how well these estimates represent the general population.

To meet this gap, we performed a longitudinal HEV serosurvey among a geographically random population-based sample of children and young adults residing in Kathmandu and Kavre districts. The aim was to gain a better understanding of the geographic distribution of HEV, characterize the incidence, and investigate risk factors related to exposure.

## Methods

### Ethical considerations

The study protocol was reviewed and approved by the Nepal Health Research Council and Ethics Review Board of Dhulikhel Hospital Kathmandu University Hospital School of Medical Sciences and Stanford University. Written informed consent was obtained from all participants or their parents or legal guardians in the case of minors age <18. In addition, we obtained written assent from children between the ages of 15 and 17. The study was conducted following the principles of the Declaration of Helsinki.

### Overview

We conducted a representative, population-based longitudinal cohort study in urban (Kathmandu) and peri-urban (Kavre) areas of Nepal to characterize community-level HEV incidence. We enrolled geographically random sample of individuals aged 0–25 years from the catchment areas of Kathmandu Medical College in Kathmandu and Dhulikhel Hospital in Kavrepalanchok, Nepal from Feb 2019 to Apr 2021 [16,17]. Participants were followed up three times, approximately 3 months, 6 months, and 12 months after initial enrollment and consent. During enrollment, relevant demographic data including age, sex, socioeconomic status, were collected.

### Sampling method

We employed a systematic random sampling strategy within our defined catchment areas. We randomly selected grid clusters, enumerated all households within each cluster, and then randomly selected participants based on age stratification using the groups 0–4, 5–9, 10–15, and 16–25 years. Our exclusion criteria were minimal to ensure a representative sample. We only excluded individuals who were not residents of the catchment area or did not fall within our specified age range at the time of the cross-sectional survey.

### Sample collection

At each visit, we collected capillary blood samples onto filter papers (TropBio FP 05-002-12) using a finger-prick (dried blood spots; DBS). The samples were dried at room temperature for 24 hours then stored in individual plastic bags with desiccant at -20 C.

### Laboratory methods

We eluted the DBS by submerging two filled filter paper protrusions containing 20 ul of dried blood in 133 μL of 1X PBS containing 0.05% Tween buffer and incubating overnight at 4°C. We then centrifuged the tubes at 10,000xg and aliquoted the supernatant, considering this DBS eluate as a 1:10 dilution of serum.

We analyzed the samples using end-point ELISA with recomWell HEV IgG ELISA kits (Mikrogen Diagnostik), which evaluate immunoglobulin IgG against the recombinant HEV-ORF2 antigen. The Mikrogen HEV ELISA kit was chosen because it had the highest diagnostic performance among commercially available kits, with a published sensitivity of 74% and a specificity of 99% [18]. We read the ELISA plates at a wavelength of 450nm (with a reference wavelength of 690nm) using a Bio-Rad iMark ELISA plate reader to produce quantitative optical density (OD) values.

### Statistical analysis

**Fig 1:**
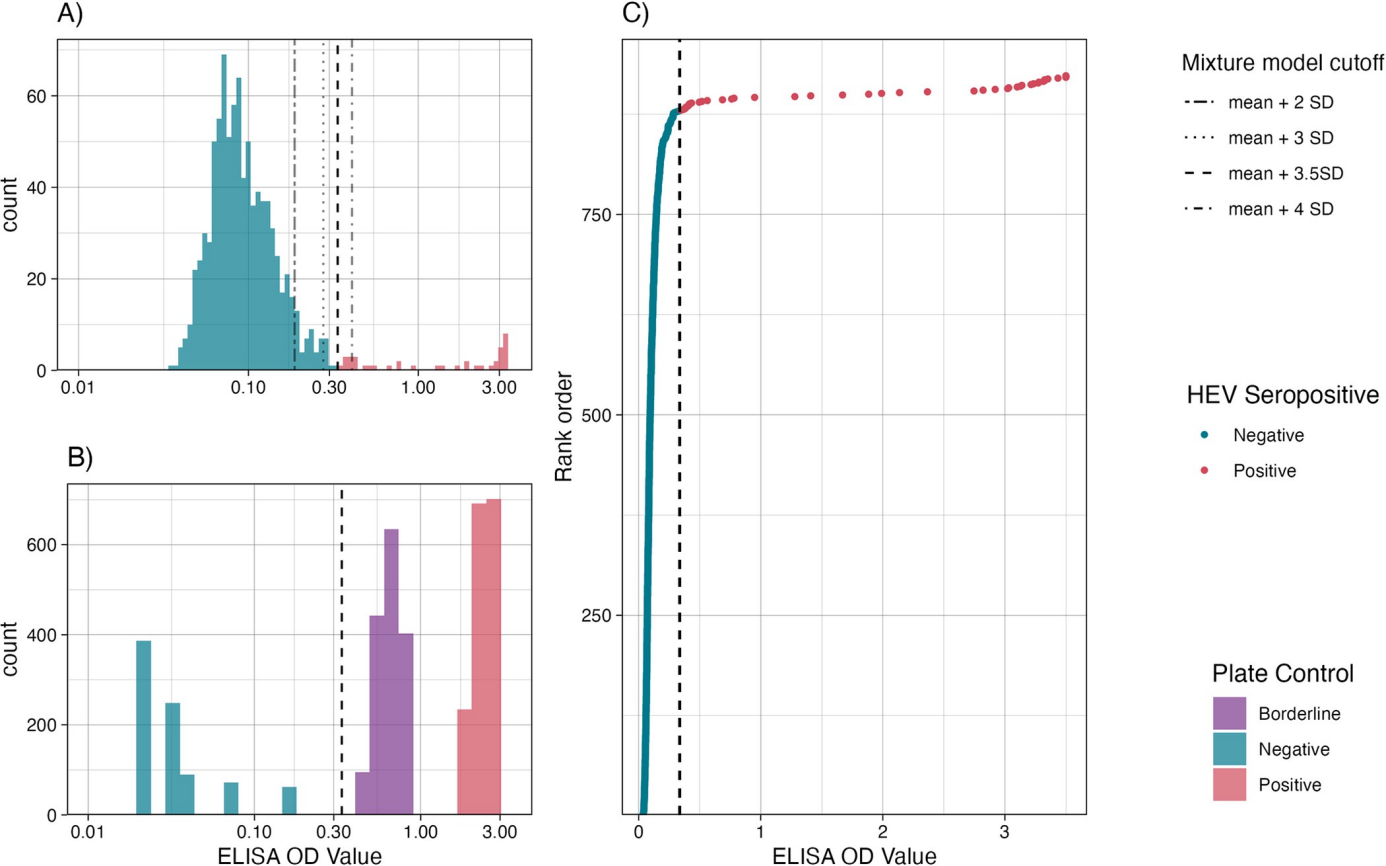
Cutoff identification methods. A) Histogram of quantitative od values among the entire population (N = 923) at the baseline study visit. Each vertical line depicts the cutoff derived from the lower component mixture model fit to the log-transformed OD values plus 2,3,3.5 and 4 standard deviations. B) Histogram of quantitative ELISA OD values for positive controls (red), borderline controls (purple), and negative controls (blue) for each plate. C) The rank order of quantitative od values among the entire population (N = 923) at the baseline study visit, each dot represents and individual result. The black dashed line depicts the cutoff derived from the mixture cutoff of the mean of the lower component plus 3.5 standard deviations. The color depicts whether the sample was seropositive (red) or seronegative (blue) using the 3.5 SD cutoff.

We used three complementary approaches to identify the optimal cutoff value (Fig 1). First, we fit 2-component Gaussian finite mixture models to the log-transformed quantitative OD values for all samples at the baseline visit. We plotted the distributions along with cutoffs derived from the mean of the lower mixture component plus 2, 3, 3.5, and 4 standard deviations (SD). The mean + 3.5 standard deviations best separated the two distributions [19] (Fig 1A). Next, we evaluated this cutoff against the distributions of positive, borderline, and negative controls from the ELISA assay (Fig 1B). The mixture-model cutoff perfectly discriminated the negative control values from the borderline and positive controls (Fig 1B). Finally, we plotted the rank order of OD values from the population assay and observed the 3.5 SD mixture model cutoff marked the inflection point in quantitative values (Fig 1C). We conducted a sensitivity analysis for the seroprevalence and seroincidence rates using cutoffs at the mixture model mean plus, 2, 3, and 4 standard deviations.

We calculated seroprevalence as the percentage of samples that were equal to or above the cutoff (seropositive) at each sampling interval. To describe how seropositivity changes over age, we fit generalized additive models with a cubic spline for age [20]. We computed simultaneous confidence intervals using a parametric bootstrap of the variance-covariance matrix of the fitted model parameters [21]. To investigate variables associated with seropositivity at baseline, we used mixed-effect binomial logit models with a random effect for the community [22].

We defined incident seroconversions or reversions as a change in IgG across the seropositivity cutoff between sampling intervals. We calculated seroincidence, defined as the number of new seroconversions per 1000 person-years, by dividing the number of individuals who seroconverted by the mid-point of person-time at risk between measurements. Similarly, we calculated the seroreversion rate as the number of individuals who seroreverted by the mid-point of person-time at risk between measurements.

We compared the seroconversion rate to the seroincidence rate derived from the cumulative hazard of the age-dependent seroprevalence at the final study visit. We fit a generalized linear model to the seropositivity conditional on age with a complementary log-log link and estimated seroincidence from the model’s intercept term [23,24]. This approach assumes that antibody responses do not wane after exposure (ie no seroreversion) and that seroincidence is constant over time.

We used negative binomial mixed-effects regression models to explore the influence of various predictors on seroincidence, including area, gender, income level, water source, and frequency of eating outside, while adjusting for age. Each predictor was individually incorporated into the model, which also accounted for repeated measurements on the same individual across different visits and individual-level variability. An offset was introduced to adjust for person-time.

We conducted a geospatial analysis to visualize the distribution of HEV seropositive individuals and seroconversions across the study area. GPS coordinates were collected in RedCap and plotted using the `ggmap' in R with stadiamaps for the baselayer. We used kernel density estimation to calculate spatial density by placing a Gaussian kernel over each data point and summing the contributions of all kernels at each location on the map.

## Results

We collected a total of 2513 dried blood samples from 923 study participants between February 2019 to April 2021. Out of 923 study participants, 401 were from Kathmandu, 197 from Panauti (Kavre), 167 from Banepa (Kavre), 85 from Dhulikhel (Kavre) and 73 from Panchkhal (Kavre). The median age of participants was 11 years (Inter Quartile Range [IQR]: 5.6–17). 21 participants were less than two years old, and the youngest age was 10 months old. Among children <2, 5 (23.8%) were exclusively breastfed, and 14 (66.7%) were fed both breastmilk and other foods and drinks. 52.2% (482/923) of participants were male. The median number of study visits completed was 3 (IQR 2–3); 229/923; 65% (603/923) of participants had 3 or more study visits while 18.2% 168/923 had just 1 study visit and 16.4% 152/923 had 2 study visits (Table 1).

**Table 1:** Demographic Characteristics of enrolled individuals.

	Banepa (N = 167)	Dhulikhel (N = 85)	Kathmandu (N = 401)	Panauti (N = 197)	Panchkhal (N = 73)	Overall (N = 923)
**Age, in years**						
Median (IQR)	10 (5.3–16)	9.5 (5.4–15)	12 (6.0–18)	11 (4.8–17)	10 (5.7–16)	11 (5.6–17)
Min/Max	1.2–27	1.1–25	0.90–27	0.90–27	1.3–24	0.90–27
**Gender**						
Female	82 (49.1%)	41 (48.2%)	183 (45.6%)	92 (46.7%)	33 (45.2%)	431 (46.7%)
Male	83 (49.7%)	43 (50.6%)	214 (53.4%)	102 (51.8%)	40 (54.8%)	482 (52.2%)
Missing	2 (1.2%)	1 (1.2%)	4 (1.0%)	3 (1.5%)	0 (0%)	10 (1.1%)
**Number of study visits**						
Median (IQR)	3.0 (3.0–4.0)	4.0 (3.0–4.0)	2.0 (1.0–3.0)	3.0 (2.0–4.0)	3.0 (3.0–3.0)	3.0 (2.0–3.0)
Min/Max	1.0–4.0	1.0–4.0	1.0–4.0	1.0–4.0	1.0–4.0	1.0–4.0
**Monthly income, Nepalese rupees**						
< 15000	11 (6.6%)	13 (15.3%)	34 (8.5%)	30 (15.2%)	12 (16.4%)	100 (10.8%)
15000–30000	47 (28.1%)	26 (30.6%)	116 (28.9%)	94 (47.7%)	29 (39.7%)	312 (33.8%)
30000–50000	49 (29.3%)	23 (27.1%)	119 (29.7%)	33 (16.8%)	13 (17.8%)	237 (25.7%)
>50000	17 (10.2%)	8 (9.4%)	55 (13.7%)	9 (4.6%)	6 (8.2%)	95 (10.3%)
Missing	43 (25.7%)	15 (17.6%)	77 (19.2%)	31 (15.7%)	13 (17.8%)	179 (19.4%)
**Primary water source**						
Municipal	88 (52.7%)	75 (88.2%)	116 (28.9%)	34 (17.3%)	13 (17.8%)	326 (35.3%)
Bottled	33 (19.8%)	0 (0%)	210 (52.4%)	0 (0%)	3 (4.1%)	246 (26.7%)
Ground	5 (3.0%)	2 (2.4%)	27 (6.7%)	55 (27.9%)	40 (54.8%)	129 (14.0%)
Private	4 (2.4%)	0 (0%)	36 (9.0%)	0 (0%)	0 (0%)	40 (4.3%)
Surface	35 (21.0%)	7 (8.2%)	7 (1.7%)	105 (53.3%)	17 (23.3%)	171 (18.5%)
Missing	2 (1.2%)	1 (1.2%)	5 (1.2%)	3 (1.5%)	0 (0%)	11 (1.2%)
**Treat water before drinking**						
No	65 (38.9%)	58 (68.2%)	190 (47.4%)	109 (55.3%)	50 (68.5%)	472 (51.1%)
Yes	100 (59.9%)	26 (30.6%)	206 (51.4%)	85 (43.1%)	23 (31.5%)	440 (47.7%)
Missing	2 (1.2%)	1 (1.2%)	5 (1.2%)	3 (1.5%)	0 (0%)	11 (1.2%)

**Fig 2:**
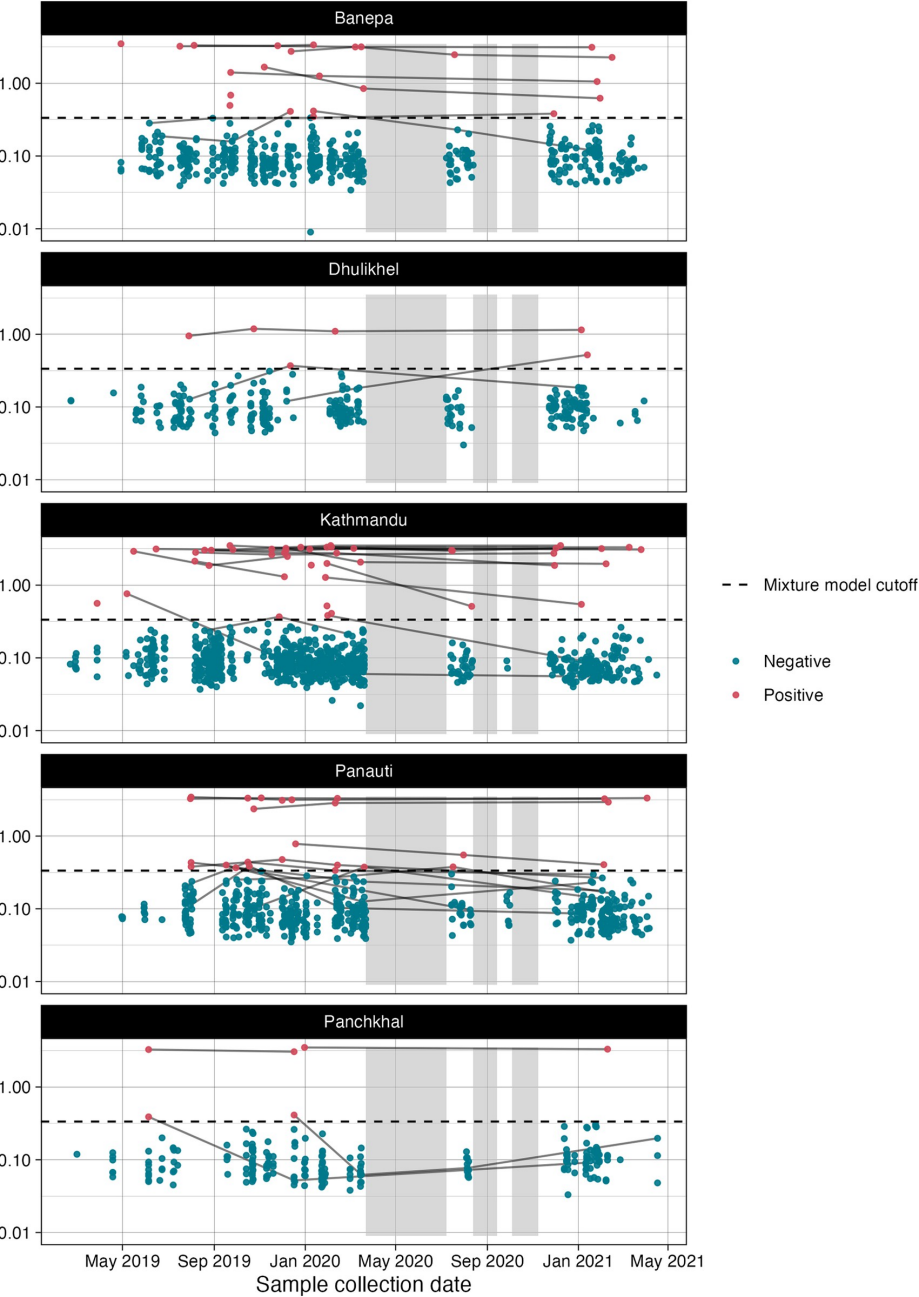
Quantitative anti-HEV IgG antibody responses over time by study site location. The dashed line indicates the mixture model cutoff. Each point represents the quantitative antibody response colored by whether it was positive (red) or negative (blue). Solid gray lines connect individual participant’s samples over time. The grey boxes indicate when sampling was paused due to COVID-19 movement restrictions.

**Fig 3:**
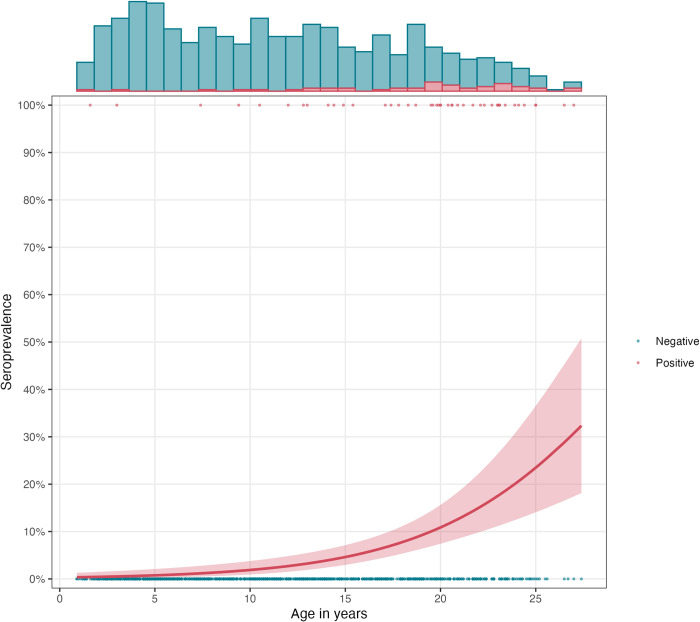
Seroprevalence as a function of age at the baseline study visit. Each point represents an individual antibody response that is positive (red) or negative (blue). The age-dependent seroprevalence curve is fit using generalized additive models with a cubic spline for age and simultaneous confidence intervals using a parametric bootstrap of the variance-covariance matrix of the fitted model parameters.

Across all visits, 106 samples (53 individuals) were seropositive for HEV (Fig 2). At the baseline visit, the crude seroprevalence was 4.8% (44/923) (Table 2). There were 9 incident seroconversions over 822.8 years of person-time, yielding a crude seroincidence rate of 10.9 (95% CI 5–20.8) per 1000 person-years (Table 3). Both seroprevalence and seroincidence increased with age. Among 0 to 5 year olds, the seroprevalence was 1.0% (2/199), increasing to 11.1% (33/297) in the 15 to 25-year-old age group (p = 0.001; see Table 2 and Fig 3). The seroincidence rate rose from 0 (95%CI: 0–32) per 1000 person-years for 0 to 5 year-olds to 9.0 (95%CI: 1–32.4) for 5 to 10 year-olds, 14.0 (95%CI: 2.9–40.8) for 10 to 15 year-olds, and 14.8 (95%CI: 4.0–37.9) for 15 to 25 year-olds (Table 3). The age-adjusted seroincidence was similar among females (10.3, 95% CI 2.8–26.3) compared to males (11.6, 95% CI 3.8–27.2) (Table 3). Results of the sensitivity analysis of the seroprevalence and seroincidence with different cutoffs are presented in the supplemental information (Table A in S1 Text). The overall seroincidence rate estimated from prospective longitudinal seroconversions was higher than the rate estimated from cumulative hazard of the age-dependent seroprevalence at final study visit (Table B in S1 Text).

**Table 2:** HEV Seroprevalence at baseline visit.

	**N()**	**N seropositive**	**Crude seroprevalence**	**Modeled seroprevalence (95% CI)**	**p value**
Overall	923	44	4.8%		
**Age, categorical**					
0-<5	199	2	1.0%	1.0% (0.3–3.9)	
5-<10	204	2	1.0%	1.0% (0.2–3.8)	0.980
10-<15	223	7	3.1%	3.1% (1.5–6.4)	0.151
15–25	297	33	11.1%	11.1% (8.0–15.2)	0.001
City/town*					
Banepa	167	10	6.0%	3.5% (1.7–7.3)	Ref
Dhulikhel	85	1	1.2%	0.7% (0.1–5.3)	0.139
Kathmandu	401	19	4.7%	2.3% (1.2–4.1)	0.279
Panauti	197	10	5.1%	2.7% (1.3–5.7)	0.583
Panchkhal	73	4	5.5%	3.6% (1.3–9.9)	0.973
Gender*					
Female	431	19	4.4%	2.4% (1.3–4.3)	Ref
Male	482	24	5.0%	2.7% (1.5–4.6)	0.725
Household monthly income, Nepalese rupees*					
<30000	412	10	2.4%	1.4% (0.7–2.9)	Ref
>30000	332	26	7.8%	4.3% (2.5–7.4)	0.003
Primary water source*					
Municipal	326	11	3.4%	1.9% (0.9–3.9)	Ref
Bottled	246	17	6.9%	3.6% (1.9–6.6)	0.119
Ground	129	5	3.9%	2.3% (0.9–5.8)	0.760
Private	40	1	2.5%	0.8% (0.1–6.3)	0.432
Surface	171	9	5.3%	2.9% (1.3–6.3)	0.368
Household treats drinking water*					
No	472	18	3.8%	2.1% (1.2–3.8)	Ref
Yes	440	25	5.7%	3.0% (1.7–5.1)	0.286

Note

*Mixed effect models adjusted for age with a random effect for city/town

**Table 3:** HEV Seroincidence.

			Seroincidence rate per 100,000 person-years		
	Incident seroconversions	person-years	Seroconversions/person-time	Modeled seroincidence*	p-value*
Overall	9	822.8	10.9 (5.0–20.8)	10.3 (5.3–19.7)	
**Age, categorical**					
0-<5	0	115.2	0.0 (0.0–32.0)	0.00 (0.00–0.31)	
5-<10	2	222.7	9.0 (1.1–32.4)	8.73 (2.19–34.89)	Ref
10-<15	3	214.7	14.0 (2.9–40.8)	13.46 (4.34–41.71)	0.019
15–25	4	270.2	14.8 (4.0–37.9)	13.08 (4.91–34.84)	0.019
City/town*					
Banepa	2	170.5	11.7 (1.4–42.4)	10.5 (2.6–42.6)	Ref
Dhulikhel	2	106.9	18.7 (2.3–67.6)	17.1 (4.2–69.2)	0.625
Kathmandu	1	247.6	4.0 (0.1–22.5)	3.4 (0.5–24.5)	0.355
Panauti	4	208.9	19.1 (5.2–49.0)	16.8 (6.1–46.3)	0.588
Panchkhal	0	89.0	0.0 (0.0–41.4)		1.000
Gender*					
Female	4	389.8	10.3 (2.8–26.3)	8.9 (3.2–24.7)	Ref
Male	5	429.4	11.6 (3.8–27.2)	10.3 (4.2–25.5)	0.831
Household monthly income, Nepalese rupees*					
<30000	3	381.6	7.9 (1.6–23.0)	7.5 (2.4–23.3)	Ref
>30000	3	283.5	10.6 (2.2–30.9)	9.4 (3.0–30.0)	0.779
Primary water source*					
Municipal	2	320.7	6.2 (0.8–22.5)	5.6 (1.4–22.8)	Ref
Bottled	1	157.7	6.3 (0.2–35.3)	5.3 (0.7–38.2)	0.958
Ground	1	138.0	7.2 (0.2–40.4)	6.5 (0.9–46.8)	0.903
Private	0	26.1	0.0 (0.0–141.3)		1.000
Surface	5	176.7	28.3 (9.2–66.0)	24.8 (10.0–61.5)	0.076
Household treats drinking water*					
No	5	442.4	11.3 (3.7–26.4)	10.1 (4.1–24.9)	Ref
Yes	4	376.8	10.6 (2.9–27.2)	9.1 (3.3–25.3)	0.879

Note

*Mixed effect poisson model adjusted for age and repeated measures

Among the 44 individuals seropositive at baseline and 9 the incident seroconversions, there were 15 seroreversion events, yielding a seroreversion rate of 28.3% (15/53). The seroreversion rate over 33.57 person-years of observation time was 446.8 (95% CI 250.1–736.9) per 1000 person-years. The seroreversion rate decreased with age and was highest among young children less than 10 years old with 4 seroreversions over 1.87 person-years and a rate 2136 per 1000 person-years. The seroreversion rates by age are presented in the supplemental information (Table C in S1 Text).

Individuals residing in households with a monthly income exceeding 30,000 Nepalese rupees had a HEV seroprevalence of 4.3% (95% CI 2.5–7.5) compared to 1.4% (95% CI 0.7–3.0) for those in households earning below 30,000 Nepalese rupees (p = 0.003). The age-adjusted seroincidence rate for the higher income group was 9.4 (95% CI 3.0–30.0) per 1000 person-years, and 7.5 (95% CI 2.4–23.3) for the lower income group.

HEV seroprevalence estimates did not vary markedly according to drinking water source, with a seroprevalence of 2.9% (95% CI 1.3–6.3) among individuals whose primary drinking water source was surface water, 3.6% (95% CI 1.9–6.6) for bottled water, 1.9% (95% CI: 0.9–3.9) for municipal water, 2.3% (95% CI: 0.9–5.8) for groundwater and 0.8% (95% CI: 0.1–6.3) for a private water company. However, the HEV seroincidence rate among individuals drinking surface water was higher than the other groups at 24.8 (95% CI: 9.9–61.5) per 1000 person-years compared to 5.6 (95% CI: 1.4–22.8) for municipal water (p = 0.076). Of the 9 incident seroconversions, 5 reported drinking surface water, and of these, 3 did not treat, 1 treated sometimes (<50% of the time), and 1 reported always treated water by boiling. However, when aggregated there were no differences in HEV seroprevalence or seroincidence according to whether the household treated their drinking water or not. Detailed seroprevalence and seroincidence are provided in Tables 2 and 3.

**Fig 4:**
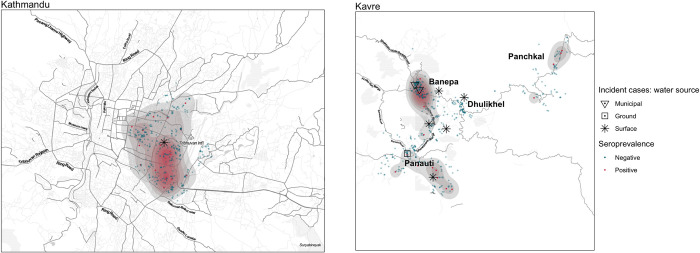
HEV Seroprevalence at baseline and prospective seroconversions across the enrollment areas (Banepa, Panauti, Dhulikhel, Panchkhal: Kavre and Kathmandu) and over the enrollment period of Feb 2019 to Apr 2021. The colored points represent seropositive (red) or seronegative (blue) participants. The black shapes indicate incident seroconversions, with a different shape for each water source. The opacity of the red-shaded area reflects the kernel density estimation. This map was created in R using ggmap. The base layer was created by Stadia Maps (stadiamaps.com) using Stamen design (stamen.com) and OpenStreetMap (openstreetmap.org/copyright).

HEV seroprevalence and seroincidence were not geographically uniform. The seropositive cases are geospatially represented in Fig 4, revealing potential clusters in both Kavre and Kathmandu districts. In Kavre district, seroprevalence was highest in Banepa 3.5% (95% CI 95% CI: 1.7–7.3) and Panchkal 3.6% (95% CI 1.3–9.9). Within Kathmandu, seropositive cases were centered in south-west of Tribhuvan airport, the one incidence seroconversion was also in this area. In Kavre, seroconversions were centered near Banepa (Fig 4).

## Discussion

This representative longitudinal serosurvey reveals ongoing HEV exposure in the Kathmandu Valley of Nepal. With a total of 2513 dried blood samples collected from 923 children and young adults, we observed a seroprevalence of 4.8% and a seroincidence rate of 10.9 seroconversions per 1000 person-years. These findings are consistent with the growing body of evidence suggesting that HEV is endemic in many parts of Nepal [11,12,14,15,25].

HEV seroprevalence and seroincidence both increase with age. Among those aged 15–25, 1 in 10 participants had evidence of HEV IgG exposure. This age group is particularly significant as it encompasses women of childbearing age, where the risks of HEV complications and mortality peak [3]. These findings align with other studies in Bangladesh [7], Nepal [25] and Laos [26], where seroprevalence increased with age. However, these other studies were limited in their ability to disentangle age and cohort effects. It’s possible that seroprevalence among older ages reflected higher periods of exposure in the past. In this study, by measuring incident seroconversions, we were able to characterize a higher risk of exposure with age. These findings align with two cohort from Bangladesh, which also found the seroincidence of HEV increased with age [27,28].

The seroreversion rate we estimated here (40.1 per 100 person-years) is higher than what was reported from Bangladesh (15 per 100 person-years) [27]. The differences in seroreversion are most likely due to the younger age range of participants in this study. Indeed, the seroreversion rate in young children less than 10 years old in Bangladesh by Dighe et al. (~180 per 100 person-years) [27] was closer to what we observed in this study (210 per 100 person-years).

The clustering of seropositive cases around Banepa in Kavre and the southwestern region of Tribhuvan airport in Kathmandu, suggests possible localized outbreaks or common sources of exposure. The majority of incident seroconversions in Kavre were within five kilometers of Banepa–where a high seroprevalence was observed at baseline. This may suggest propagative transmission from a potential previous outbreak. Such clustering has been observed in other studies [29,30], and suggests a role for geographically-targeted public health interventions.

We identified water source as a potential risk factor for HEV seroincidence but not seroprevalence. Individuals consuming surface water had more than four times the seroincidence rate of HEV compared to those relying on other water sources. The prevalence of consuming surface water among individuals who seroconverted (5/9, 55.6%) was higher than in the general population (179/923, 19.3%) but not in Panauti (105/197, 53.3%) where the majority of seroconversions occurred. However, when restricting just to within Panauti, 3 of 4 individuals who seroconverted consumed surface water, and the other consumed groundwater. Our findings aligns with previous studies that have identified contaminated water as a primary transmission route for HEV [29,30].

While we observed a higher HEV seroprevalence in households with a reported higher income, the seroincidence rate was similar. Possible explanations for the higher seroprevalence among higher-income households include a higher frequency of eating outside the house and living in more urban settings with higher population density. There was also a significant amount of missing data for household income question, likely due to sensitivity around reporting.

Variability in HEV seroprevalence estimates across studies, even within the same region, is well-documented [31]. Some of these discrepancies can be attributed to methodologies that overlook the age-dependent nature of seroprevalence and the role of seroreversion. Ignoring the waning of antibodies underestimates seroprevalence and seroincidence when derived from age-dependent seroprevalence estimates [32]. In our study, the seroincidence measured from longitudinal seroconversions was three times higher than the seroincidence rate derived from the age-dependent cross-sectional seroprevalence that ignored antibody decay. Dighe et al. also found that the HEV seroincidence rate using longitudinal seroconversions was 5 times that of the age-dependent seroprevalence when ignoring seroreversions [27]. Together, these findings underscore the need to factor in seroreversion when reporting HEV seroprevalence and seroincidence rates.

Several limitations are important to acknowledge when interpreting these results. First, this study leveraged a cohort designed to characterize enteric fever burden, which did not include individuals above 25 years of age. While this restricted our ability to comment on the HEV burden in older age groups, prior research has indicated that the most significant HEV burden is among young adults [7,25]. Future studies are needed to characterize HEV seroincidence in older ages in the Kathmandu Valley region. Second, our use of dried blood samples, though logistically advantageous, might have increased the limit of detection our serological assays. A recent study comparing anti-HEV IgG antibody responses in dried blood spots a sensitivity of 81% and a specificity of 97% comkpared to to *fresh serum* [33]. Given this reduced sensitivity, our findings may underestimate the true HEV seroprevalence and seroincidence by up to 20%. However, another study in Bangladesh found OD values for measuring Anti-HEV IgG response from DBS were equivalent to plasma when stored properly [34]. Third, with only 9 seroconversion events, we were underpowered to detect effect sizes less than 2. Fourth, when evaluating the risk associated with water sources, it is likely that individuals consumed water from multiple sources, yet we were only able to capture information about the primary source. Finally, our household sampling strategy could have inadvertently omitted migrant populations and residents of informal settlements. Such populations, often faced with subpar water, sanitation, and hygiene conditions, could potentially have a higher HEV seropositivity rate, potentially biasing our findings towards the null.

In summary, our study demonstrates that HEV is endemic in Nepal and that exposure increases with age. These insights emphasize the need for targeted public health strategies such as vaccination and improved water and sanitation infrastructure.

## Supporting information

S1 Text Supplemental tables. (DOCX)
