# Supplementary material for: Hepatitis E virus in the Kathmandu Valley: Insights from a representative longitudinal serosurvey
Source: PLoS Negl Trop Dis. 2024 Aug 5;18(8):e0012375. doi: 10.1371/journal.pntd.0012375 (PMC11326703; doi:10.1371/journal.pntd.0012375)
Supplement: S1 Text — (DOCX) [file pntd.0012375.s001.docx]

**SUPPLEMENTAL MATERIAL**

**Hepatitis E Virus in the Kathmandu Valley: Insights from a Representative Longitudinal Serosurvey**

**Authors**: Nishan Katuwal^1,2^, Melina Thapa^1^, Sony Shrestha^1^, Krista Vaidya^1,3^, Isaac I Bogoch^4^, Rajeev Shrestha^1,2^, Jason R Andrews^5^, Dipesh Tamrakar^1,2,6¶^, Kristen Aiemjoy^3,7¶^*

***Corresponding Author:**

Email: [kaiemjoy@ucdavis.edu](mailto:kaiemjoy@ucdavis.edu)

**Table A. Sensitivity analysis of seroprevalence and seroincidence rate to different cutoff values**

|  | **Baseline** | | | **Overall** | | |
| --- | --- | --- | --- | --- | --- | --- |
| **Cutoff** | **N seropositive** | **Total N** | **Seroprevalence** | **Incident Cases** | **Person-years** | **Seroincidence rate per 1000 PYs** |
| +2 SD | 97 | 923 | 10.5% | 61 | 800.2 | 76.23 |
| +3 SD | 53 | 923 | 5.7% | 15 | 817.2 | 18.34 |
| +4 SD | 38 | 923 | 4.1% | 4 | 825.3 | 4.84 |

**Table B. Seroincidence derived from the age-dependent seroprevalence**

|  | **Seroincidence rate per 1000 person-years** |
| --- | --- |
| Overall | 3.4 (2.5-4.7) |
| **Gender** | |
| Female | 6.8 (2.8-16.3) |
| Male | 3.7 (1.4-9.9) |
| **Age, categorical** | |
| 0-<5 | 3.0 (0.8-12.0) |
| 5-<10 | 1.4 (0.3-5.4) |
| 10-<15 | 2.6 (1.2-5.4) |
| 15-25 | 6.0 (4.3-8.5) |
| **City/town*** | |
| Banepa | 5.8 (3.1-10.7) |
| Dhulikhel | 1.1 (0.2-7.8) |
| Kathmandu | 4.0 (2.6-6.3) |
| Panauti | 4.6 (2.5-8.6) |
| Panchkhal | 5.1 (1.9-13.6) |

**Table C. Seroreversions by age category**

|  | **Seroreversions** | **Person-years** | **Seroreversion rate per 1000 person-years** |
| --- | --- | --- | --- |
| **Overall** | | | |
| Overall | 15 | 37.47 | 400.4 (224.1-660.3) |
| **Age, categorical** | | | |
| 0-<5 | 1 | 0.49 | 2027.8 (51.3-11298.1) |
| 5-<10 | 3 | 1.38 | 2174.8 (448.5-6355.6) |
| 10-<15 | 5 | 4.34 | 1152.9 (374.3-2690.4) |
| 15-25 | 6 | 31.26 | 192.0 (70.4-417.8) |
